# Evaluation of Lay Support in Pregnant women with Social risk (ELSIPS): a randomised controlled trial

**DOI:** 10.1186/1471-2393-12-11

**Published:** 2012-02-29

**Authors:** Sara Kenyon, Kate Jolly, Karla Hemming, Lucy Ingram, Nicola Gale, Sophie-Anna Dann, Jacky Chambers, Christine MacArthur

**Affiliations:** 1 School of Health and Population Sciences, University of Birmingham, Edgbaston, Birmingham B15 2TT, UK; 2 Heart of Birmingham Teaching PCT, Bartholomew House, 142 Hagley Road, Edgbaston, Birmingham B16 9PA, UK

## Abstract

**Background:**

Maternal, neonatal and child health outcomes are worse in families from black and ethnic minority groups and disadvantaged backgrounds. There is little evidence on whether lay support improves maternal and infant outcomes among women with complex social needs within a disadvantaged multi-ethnic population in the United Kingdom (UK).

**Method/Design:**

The aim of this study is to evaluate a lay Pregnancy Outreach Worker (POW) service for nulliparous women identified as having social risk within a maternity service that is systematically assessing social risks alongside the usual obstetric and medical risks. The study design is a randomised controlled trial (RCT) in nulliparous women assessed as having social risk comparing standard maternity care with the addition of referral to the POW support service.

The POWs work alongside community midwifery teams and offer individualised support to women to encourage engagement with services (health and social care) from randomisation (before 28 weeks gestation) until 6 weeks after birth.

The primary outcomes have been chosen on the basis that they are linked to maternal and infant health. The two primary outcomes are engagement with antenatal care, assessed by the number of antenatal visits; and maternal depression, assessed using the Edinburgh Postnatal Depression Scale at 8-12 weeks after birth. Secondary outcomes include maternal and neonatal morbidity and mortality, routine child health assessments, including immunisation uptake and breastfeeding at 6 weeks. Other psychological outcomes (self efficacy) and mother-to-infant bonding will also be collected using validated tools.

A sample size of 1316 will provide 90% power (at the 5% significance level) to detect increased engagement with antenatal services of 1.5 visits and a reduction of 1.5 in the average EPDS score for women with two or more social risk factors, with power in excess of this for women with any social risk factor. Analysis will be by intention to treat.

Qualitative research will explore the POWs' daily work in context. This will complement the findings of the RCT through a triangulation of quantitative and qualitative data on the process of the intervention, and identify other contextual factors that affect the implementation of the intervention.

**Discussion:**

The trial will provide high quality evidence as to whether or not lay support (POW) offered to women identified with social risk factors improves engagement with maternity services and reduces numbers of women with depression.

**MREC number:**

10/H1207/23

**Trial registration number:**

ISRCTN: ISRCTN35027323

## Background

Maternal, neonatal and child health outcomes are worse in women from black and ethnic minority and disadvantaged groups and there are a range of factors likely to be contributing, one of these being inclusivity and engagement with services. This was an important focus of the National Service Framework (NSF) [1] on Maternity and Maternity Matters [2], both of which emphasise choice, access and continuity of care in a safe service. The last two Confidential Enquiries into Maternal Deaths and Saving Mothers' Lives made recommendations about care for vulnerable women with socially complex lives [3]. Social disadvantage, living in a poor community and being from a minority ethnic group, including asylum seekers and newly arrived refugees, were all major risk factors. Black African women, including asylum seekers and newly arrived refugees had a mortality rate nearly six times higher than White women [3].^. ^Clearly maternal death is rare but it is well documented that women from these vulnerable groups book for antenatal care later, make fewer visits, experience greater pregnancy morbidity and have a higher risk of adverse fetal and child health outcomes. A recent UK national cohort study [4] found severe maternal morbidities were significantly more common among women from black African and Caribbean and Pakistani ethnic groups than in White women. The authors suggested that these differences may be due to pre-existing medical factors or factors related to care during pregnancy, labour or birth but they are unlikely to be due to differences in age, socioeconomic or smoking status, body mass index or parity. This study further highlighted the importance of tailored maternity services and improving access to care for women of ethnic minorities. Based on the assumption that increased engagement with antenatal services will result in improved maternal and perinatal health outcomes, the maternity NSF recommended that services are proactive in engaging all women, particularly those from disadvantaged groups. This includes contact early in their pregnancy and maintenance of contact before and after birth. It notes that some women in these groups may require more support and access to social or other services, for example housing and benefits advice.

In informing the NSF, evidence was sought on how services may be organised and delivered to improve outcomes for disadvantaged groups, but little good evidence was found [5]. The National Institute for Health and Clinical Excellence (NICE) has recently published a Guideline for Models of Service Provision for pregnant women with complex social factors [6] and found little high quality evidence. One of the research recommendations was to answer the question 'Is intervention and/or family support provided by statutory and 3^rd ^sector agencies effective in improving outcomes for women and their babies?'

Additional social support during pregnancy for vulnerable groups might, on the face of it, be of possible benefit. However, a recently updated Cochrane review of 'Support during pregnancy for women at increased risk of low birth weight' (LBW) [7] found 18 RCTs and concluded that programmes offering additional social support were not associated with improvements in any perinatal outcomes. In most of the trials, however, participants were selected because they had obstetric rather than social risks for LBW and almost all support interventions were delivered by trained professionals, which may not be the most likely person to improve outcomes. The review did find an overall reduction in Caesarean section (RR 0.87, 95%CI 0.78 to 0.97), and noted that some trials found improvements in maternal psychosocial outcomes [7]. It is well documented that a reduction in maternal depression, in addition to improving maternal wellbeing, will have a beneficial effect on short and long term child outcomes [8,9].

There is some evidence on benefits of lay support in other areas of maternity care from the Cochrane review on 'Continuous Support for Women during Childbirth' [10], which suggested that the beneficial effects associated with continuous support were greater when the provider was not a member of the hospital staff. The review showed that when the providers of continuous support were members of staff, spontaneous vaginal birth (SVB) was increased. When the providers of support were not staff members SVB appeared to be further increased. This therefore adds support to the hypothesis that care may be better received when provided by lay people rather than health professionals.

Evidence from three much quoted trials by Olds in the United States, of nurse home visitation from pregnancy up to 2 years for vulnerable groups (one mainly teen, single mothers [11] and the second, young deprived African-Americans [12]) found improvements in some pregnancy-related outcomes, including greater engagement with maternity and related services, and went on to find beneficial effects in several long-term maternal and child outcomes. Only the first trial, however, found any effect on preterm delivery and birth weight and only in the small sub-groups of those aged 14-16 and of smokers [11]. The third trial by Olds also examined the effects of home visitation by lay workers but found no benefit from this type of worker [13]. Evaluation of the Old's support model of nurse home visitation is currently being undertaken throughout the UK (Family Nurse Partnership (FNP)).

At present therefore, there is little evidence on whether lay support improves maternal and infant outcomes among women with complex social needs within a disadvantaged multi-ethnic population in the UK. A recent meta-synthesis [14] into barriers to antenatal care for marginalised women in high income countries has suggested that a non-judgemental, contextually tailored antenatal service that pays attention to the specific circumstances of disadvantaged women may increase sustained access to care.

So at least in theory, care that provides individual case management including home visiting, as provided by a POW service, could be of benefit.

The aim of this study is to evaluate, by a randomised controlled trial, a POW service for nulliparous women identified as having social risk within a maternity service that is systematically assessing social risks alongside the usual obstetric and medical risks.

## Methods/Design

### Design

**Figure 1:**
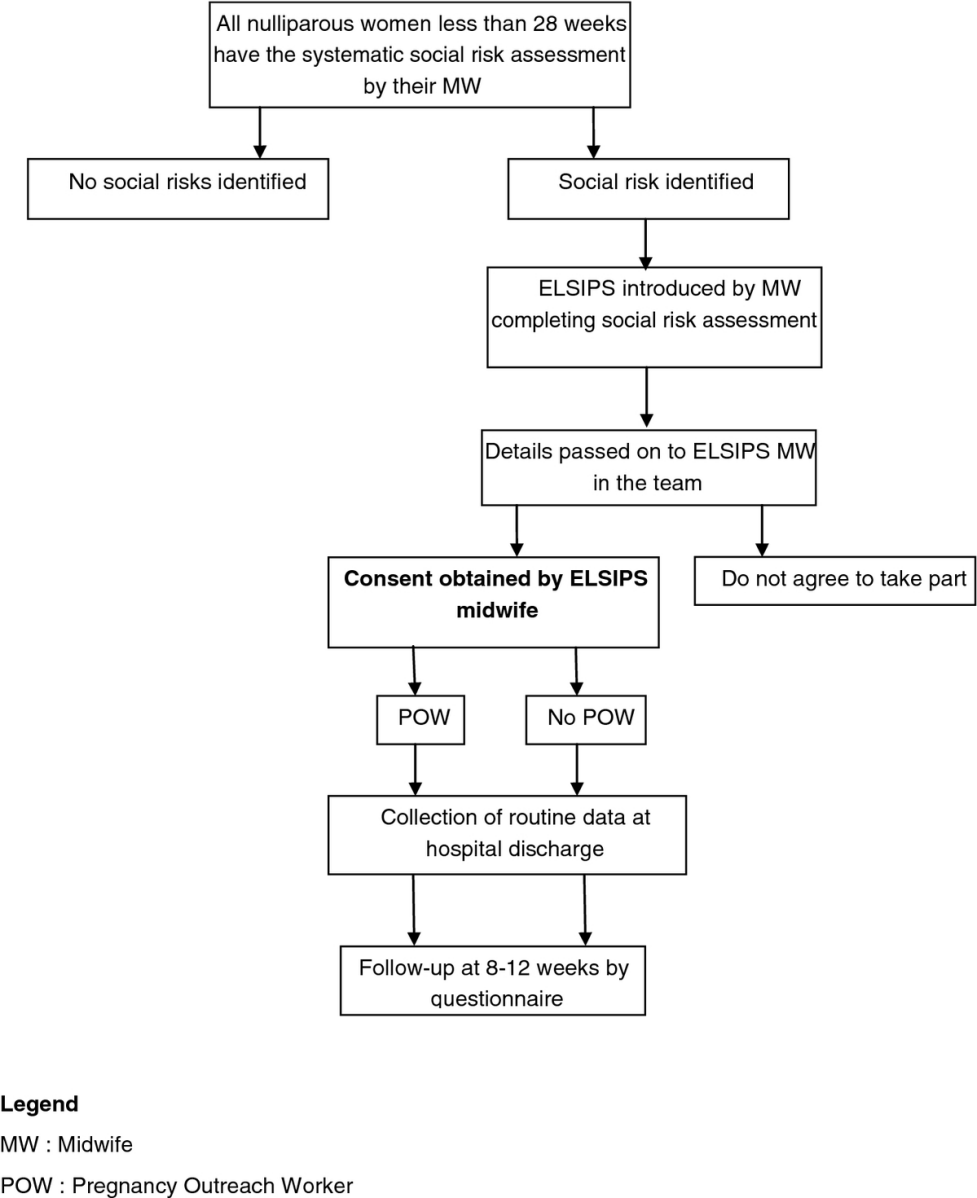
**Flow diagram to summarise allocation and contact throughout the trial**.

The study design is an individually randomised controlled trial involving three primary care trusts (PCTs) in Birmingham, with nulliparous women assessed as having social risk, randomised to standard maternity care or the addition of referral to the POW support service (See Figure 1).

### Setting and population

The POW service and its evaluation will run across the whole of Birmingham, which currently comprises three PCTs: Heart of Birmingham (HoB PCT); South Birmingham (SB PCT) and Birmingham East and North (BEN PCT). Although varying in proportion, these PCTs all include a population that has high levels of deprivation and with a variety of ethnic groups, including many recently arrived mothers, refugees and asylum seekers. For example, in HoB PCT there are 5500-6000 births each year; almost 90% of which are to women in deprived wards (Index of Multiple Deprivation (IMD) quintile 5) [15]. Only about 15% are of European ethnicity, 54% are within the South Asian group (31% Pakistani, 11% Bangladeshi and 9% Indian), 13% are African and 10% African-Caribbean. One in four mothers has themselves been born outside the UK and many are recent immigrants who have little English language skills. In SB PCT, about 50% of births occur to women in the most deprived IMD quintile of deprivation and 60% of mothers are white. BEN PCT has a population somewhere between these in terms of the proportion of women in the ethnic minority groups and levels of deprivation. In summary, women from black and ethnic minority groups with complex social risk and needs accounts for a large and increasing proportion of the maternity population cared for by the three maternity units (Birmingham Women's National Health Service (NHS) Foundation Trust, Sandwell & West Birmingham NHS Trust, and Heart of England NHS Foundation Trust) in these PCTs.

### Usual care (control)

To maximise access and engagement with maternity services the local PCTs have commissioned a new service model based on social risk assessment, alongside assessing and managing obstetric and medical risk in the usual way. To assess social risk a set of items have recently been included in the standardised maternity notes used universally in Birmingham. These are completed at the booking visit by the midwife and identify whether a woman has any of the following factors:

• UK resident for under a year.

• Difficulty with the English language, both spoken and written.

• Housing problems, such as rent arrears, temporary accommodation, registered with National Asylum Support Service (NASS) or of No Fixed Abode (NFA).

• No support from either partner or family or friend

• Woman/household member in receipt of social services support, including child protection.

• Identified benefit problem.

• Smoking.

• Drug misuse, including other's in the household.

• Alcohol misuse.

• Clinical diagnosis of past or present mental illness.

• Teen parent (under 20 years old).

• Domestic abuse.

• Body Mass Index less than or equal to 18 OR more than or equal to 35.

• Late booking (defined as booking after 18 weeks gestation).

• Did Not Attend 2 or more antenatal appointments (under 28 weeks gestation).

The intention is that systematic social risk assessment will maximise the likelihood that social risk is identified and needs are met. Midwives identifying women with social risk factors currently either signpost women to services that may be beneficial or refer them to specialised agencies or personnel. This may mean they signpost to support agencies (for example, housing or benefit offices) or refer to other agencies (for example, social services), or refer onto the specialist midwives in their Trust. These specialist midwives act as a contact point and provide specific advice and support for women experiencing problems such as domestic abuse, mental health issues or who are teenagers when they are pregnant.

### Intervention

The PCTs have also decided to provide a POW service to complement these pathways, with the intention of further increasing full engagement with care during pregnancy and postpartum, and to improve the women's social conditions. The ultimate aim of this, theoretically at least, is to improve the health of both mother and baby by increasing engagement with antenatal services, which should reduce perinatal mortality and morbidity. It is also hoped that additional support of this nature would improve women's psychological health, which in turn would have a positive impact on the child.

The POW service is in addition to standard maternity care and will not be available to nulliparous women other than within the trial. Women assessed as having social risk and randomised to the intervention group will be referred to a POW who will provide individual case management including home visiting. The purpose of the POW service is to ensure that women attend antenatal appointments and engage with required care, such as taking prescribed medication, attending scan appointments, and including making lifestyle changes, such as smoking cessation. Additionally in the postnatal period the POWs are providing breast feeding support (World Health Organization Baby Friendly Initiative) and advice about feeding and caring for the baby. The POWs also provide social support on such issues as ensuring that available benefits are obtained, housing difficulties are dealt with, mental health problems managed and overall well-being is maximised. The philosophy underlying POW support is an attempt to help women to become more able to manage problems that arise in life, that is, to enhance their general self-efficacy. The POWs receive appropriate training to National Vocational Qualification (NVQ) level 3 which is provided by 'Gateway Family Services' and have access to supervision from experts with specific skills and knowledge. Postpartum POW contact will continue until 6 weeks after birth when transfer to the Family Support Worker (FSW) would take place for those who require it.

### Eligibility

#### Inclusion criteria

• Nulliparous women < 28 weeks gestation.

• Assessed by the midwife as having specified social risk through routine systematic assessment.

Nulliparous is defined as never having given birth to a child; this will include women who have had a miscarriage/s or termination/s of pregnancy. We have chosen under 28 weeks as an inclusion criterion to give adequate time for the POW service to impact on the outcomes.

#### Exclusion criteria

One of the PCTs (SB PCT) participating in this trial is also involved in a national trial of additional support to pregnant teenagers, called the Family Nurse Partnership (FNP). The FNP intervention is health professionals providing intensive support throughout pregnancy up to 2 years after birth. We will exclude teenagers recruited to FNP, but do not expect this to greatly affect recruitment to ELSIPS. We will also exclude those under 16 years of age due to the complexity of gaining informed consent from this group.

### Recruitment and randomisation

As part of the booking visit, all women in Birmingham have a systematic social risk assessment undertaken by the midwife. Once social risk has been identified the midwife will offer the support routinely available (as part of standard care) and will discuss the additional support of the POW only available through the trial. If the woman is potentially interested in taking part, her details will be passed to the ELSIPS midwife within the community midwifery team.

Randomisation is by random permuted blocked design stratified by Trust. The randomisation lists were generated by the trial statistician (KH) and then forwarded to the University of Birmingham Primary Care Clinical Research and Trials Unit who provided a telephone randomisation service thus ensuring concealment of allocation.

### The ELSIPS midwives

All the midwives within their individual teams refer eligible women to the ELSIPS midwife. The ELSIPS midwife is responsible for obtaining informed consent and randomising the women. They are also responsible within their teams for promoting the trial and training other midwives in trial processes. The ELSIPS midwives work for a varying number of hours (3-7) per week depending on the size of their team and the number of births. They each have an agreed target for the numbers of women we expect to be recruited each month. They are trained and supported by the University of Birmingham team.

### Outcome measures

The primary outcomes have been chosen on the basis that they are linked to maternal and infant health. The two primary outcomes are engagement with antenatal care, assessed based on number of antenatal visits, and maternal depression, assessed using the Edinburgh Postnatal Depression Scale [16] (EPDS) at 8-12 weeks after birth.

### Secondary outcomes

#### Maternal outcomes will include

• length of labour (first, second and third stages),

• mode of birth (spontaneous vaginal birth, instrumental birth or caesarean section),

• perineal trauma (episiotomy, degree of laceration),

• incidence of maternal morbidity (e.g., postpartum haemorrhage, shoulder dystocia, chorionamnioitis),

• length of stay in hospital,

• engagement with other services, as required (e.g., smoking cessation service).

#### Baby outcomes are mainly markers of poor perinatal outcome

• composite outcome of adverse perinatal outcome comprising:

- perinatal mortality;

- preterm birth before 34 weeks;

- birth weight 10th centile or below;

- admission to neonatal unit

• Apgar score at 5 minutes,

• arterial *c*ord blood gases, if taken

• breastfeeding initiation rate,

• length of stay in hospital,

• oxygen at 36 weeks post conceptual age, if applicable,

• retinopathy of prematurity, if applicable,

• abnormal cerebral ultrasound prior to discharge (e.g., intraparenchymal cerebral bleed, hydrocephalus, parenchymal cysts), if applicable,

• necrotising enterocolitis (Bells Stage I, II or III), if applicable,

• culture positive sepsis requiring greater than 5 days antibiotic treatment, if applicable.

#### Longer term infant outcomes

• Routine child health assessments, including immunisation uptake and breastfeeding continuation at 6 weeks.

#### Psychological outcomes

• Self efficacy (using Pearlin and Schooler Mastery Scale [17]).

• Mother-to-infant bonding tool [18].

The detrimental impact of maternal bonding difficulties on both the emotional and cognitive development of the child and the quality of the mother-infant relationship has been well documented in the literature [19]. We have therefore chosen to evaluate mother-to-infant bonding.

### Sample size

Currently, NICE recommend, in the Antenatal Care Guideline [20], that the schedule of appointments should be determined by the function of the appointments and for nulliparous women with an uncomplicated pregnancy, they recommend that a schedule of ten visits should be adequate. The actual number of appointments attended was the subject of a survey of women's experiences of maternity care [21] carried out in 2006. This was a national survey which used a random sample of 4800 women and achieved a response rate of 63%. Women were sent a postal questionnaire three months after birth. Nulliparous women reported they attended a mean of 10.9 (standard deviation [SD] 6) antenatal appointments (Table 1).

**Table 1:** Estimated frequency of the events which make up the primary outcomes

Outcome	Average	Source of information
Number of antenatal visits by nullips (consultant and midwife)	Average number of visits 10.9 (SD 6)	Recorded Delivery [21]

Edinburgh Postnatal Depression Scale (EPDS)	Average score5-7 (SD 6)	Psychosocial and psychological interventions for preventing postpartum depression (Cochrane review) [22]

There is some evidence relating to number of antenatal visits and perinatal outcomes. A systematic review [23] found that in settings where the number of visits is already low, reduced visits programmes of antenatal care for low risk women are associated with an increase in perinatal mortality compared to standard care, although admission to neonatal intensive care may be reduced.

An observational study explored the relationship between the number of antenatal visits made by 17,765 British women and adverse perinatal outcomes [24]. No consistent relationship between admission to neonatal unit or perinatal mortality and number of antenatal visits was found. A significant positive relationship was found between number of antenatal visits and Caesarean section, and low birth weight (less than 2500 g) was positively associated with number of visits for nulliparous women but not for parous women. More recently, a cohort study from Finland found under-attending free antenatal care was associated with adverse pregnancy outcomes [25]. Logistic regression analyses found there were significantly more low birthweight infants in the under and non-attenders, with more fetal and neonatal death.

Estimates of the baseline Edinburgh postnatal depression score are taken from the controls of a Cochrane systematic review of Psychosocial and psychological interventions for preventing postpartum depression [22]. It is well documented that a reduction in maternal depression, in addition to improving maternal wellbeing, will have a beneficial effect on short and long term child outcomes and it is plausible that social support provided by the POWs could reduce the numbers of women becoming depressed. Studies have shown that depressed mothers are more likely to demonstrate impaired maternal-infant interactions and negative perceptions of infant behaviour [8]. Children of depressed mothers are more likely to suffer a range of adverse outcomes, including insecure attachment, behavioural problems, cognitive developmental deficits and difficulties in emotional functioning, some of these continuing into adolescence [9,26-29].

A sample size of 421 per arm would provide 90% power (at the 5% significance level) to detect a reduction of 1.5 in the average EPDS score from say 7 to 5.5, and would provide greater than 90% power to detect increased engagement with antenatal services of either 1.5 or 2 visits (Table 2). This calculation has also allowed for 20% drop-out or loss to follow-up.

**Table 2:** Estimated sample size calculations for number of antenatal visits

Average number of antenatal visits	Average number in intervention arm	Difference	Power	Sample sizeper arm
8.9	10.9	2	90%	190

9.4	10.9	1.5	90%	337

Following a successful six month pilot in which 475 women were recruited a revision to the sample size was agreed. Prior to the pilot there was no data on the extent of the social risk factors amongst women and data from the pilot showed that 36% of the women recruited had one social risk factor. It was agreed to power the study to detect the pre-specified differences in the primary outcomes in the sub group of women with two or more social risk factors, which lead to an increase in the sample size to 658 women per arm. It is anticipated that this sample size of 1316 will be obtained by 31st December 2011.

### Statistical analysis

We will calculate the mean number of visits and mean EPDS for each arm and compare differences using the *t*-test or other appropriate non-parametric test. Variations will be explored by pre-specified sub-group comparisons. Variations in participant baseline characteristics between intervention and control groups will also be explored, and if appropriate, variations in participant characteristics adjusted for using generalised linear models with appropriate consideration of strata of randomisation.

For secondary continuous outcomes, variations between control and intervention groups will be investigated using the *t*-test and, if necessary, covariate adjustment made using generalised linear models (to include strata affects); for secondary binary outcomes, differences will be compared using chi-squared tests and, if necessary, logistic regression. For all analyses, assumptions of various tests and models will be explored and non-parametric tests used if required.

All analyses will be by intention to treat and 95% confidence intervals will be quoted throughout. Missing covariate and outcome data will be examined. Reasons for withdrawal, lack of participation and any reasons for non-compliance will be documented and explored. Complete case and available case analyses will be completed in the first instance. If the amount of missing data is not insignificant, then multiple imputation will be used to evaluate sensitivity to the missing completely at random assumption, and inferences compared to those under the lesser missing at random assumption.

Pre-specified sub-group comparisons will be according to number of social risks (1 social risk or 2 or more social risks) identified and gestation at recruitment (< 12 weeks, 12-19 + 6 weeks, 20-27 + 6 weeks). These pre-specified subgroup comparisons will be for the primary outcomes and the more clinically important secondary outcomes (perinatal composite outcome, self-efficacy and Mother-to-infant bonding). The level of significance will at 0.05. The remaining secondary outcomes will have the level of significance at 0.01.

### Qualitative component to the ELSIPS study

We will undertake additional qualitative research in order to understand the nature of the work of the POW in more depth. This will complement the findings of the RCT, in which process information is already being collected by the POWs about the components of their work, including the frequency, venue, duration, support offered, additional social risk disclosure and referrals to other agencies.

There are two aims of this work:

1. It will allow us to ensure that the components and process of the intervention itself (the relationship and support offered by the POW) are fully understood, and help future policy makers determine whether the intervention might need to be adapted to reflect their population and health system.

2. It may help to identify aspects of the intervention that are particularly successful and areas that would benefit from future redesign or efficiency/quality improvement work.

Qualitative approach: A grounded theory approach [30] will be taken to this part of the study, in order to understand the nature of the POWs work from their perspective. We have chosen to avoid formal interviews of the POWs about their work as this tends to reproduce the 'theory' about the role, idealised accounts and retrospective explanations about action [31,32] and is therefore less likely to be able to fully uncover any disjunction between the process information collected about their work and the complexity of daily practice. We have selected shadowing [33], a form of focused ethnography, to understand the nature and content of the daily work of the POWs in a naturalistic environment. This enables observations of action-incontext that can be triangulated with informal reflective discussions with POWs on aspects of their work that have been observed.

Data collection method: Two researchers (one social scientist, one clinical researcher) will singly observe POWs undertaking their daily work, including meetings with clients, until theme saturation is achieved. It is anticipated that this will take approximately 100 hours.

Sampling: Initial sampling will be purposive to include two POWs from each of the three localities covered by the service. The rationale behind this was that each locality serves a population with very different socio-demographic characteristics. We will coordinate with the POW managers to ensure that we are able to observe interactions with women at different stages in the POW service. Any further sampling will be theoretical (see analysis below).

Recruitment: Researchers will attend team meetings to inform POWs about the study. Written consent will be obtained from the POWs for the shadowing. If POWs are selected to participate, all their current clients will receive a letter informing them that their POW may be accompanied by a researcher and that they can opt out without it affecting the service they receive. Each POW will confirm orally that the client has received and understood the letter on a case-by-case basis before any meetings are observed.

Analysis: Field notes will be taken during the observation and detailed reflective accounts of the observation written up immediately following the observation. After each day of shadowing, the two researchers will meet to debrief, discuss the data and emerging themes, and to identify additional data required to elaborate the properties of emerging themes and test them. Emergent themes will be used to interrogate existing related theory in the literature and extend it.

Data anonymization and storage: Full field notes will only be available to the core qualitative analysis group (NG, LI, SK). Before dissemination, all data will be anonymized and any potentially identifying features of the clients the POWs worked with will be removed.

### Trial oversight

A Steering Committee has been formed from all those involved to monitor progress and oversight is provided by the Birmingham and Black Country Collaboration in Leadership in Applied Research and Care (CLAHRC) Steering Committee (Chair Dr Rashmi Shukla). This trial comprises of part of the work undertaken by Theme 5.

Ethical approval has been obtained from South Birmingham Ethics Committee (10/H1207/23) Approval at each site has been obtained from the local Research and Development Directorates. Participants receive an information leaflet (full and summary) and sign a consent form which are available on the website, http://www.bham.ac.uk/elsips

## Discussion

The original intention of the ELSIPS trial was to include all pregnant women. However, our detailed investigations of current social support within the PCTs have found that many multiparous women with high social need will already have been allocated a FSW through their local Children's Centre. This is because multiparous women access the Children's Centres for services for their children under 5 years old and so come into contact with the FSW on a regular basis. These FSWs are provided through local education, as well as health services funding, and many provide very similar support to a POW. The main distinction between FSWs and POWs is the FSWs do not specifically engage with nulliparous women in the antenatal period. On this basis if multiparae were included, the trial comparison may fail to find a real difference because of the dilution effect of FSWs. In addition, contamination may arise if systematically more multiparae randomised to standard care who did not already have a FSW were subsequently provided with one because of not having been allocated to a POW.

We explored powering the study using a composite primary outcome of perinatal morbidity and mortality but the substantial sample size required to show even a large difference, together with funding constraints for the POW service whilst under evaluation, have meant we have opted for the smaller sample size required for the primary outcomes of engagement with services and EPDS at 8-12 weeks after birth. Although the scientific basis for antenatal care does not appear to be as robust as it might, it is based on the assumption that engagement with services results in improved maternal and perinatal health outcomes. So, the number of visits (both consultant and midwife) attended should act as a surrogate for improved maternal and neonatal outcomes.

We have chosen to evaluate self-efficacy as one of the psychological outcomes rather than self-esteem as it is more closely related to the changes the POWs are intended to facilitate. Self-esteem is believed to reflect evaluation of one's overall self-worth, while self-efficacy is specifically concerned with evaluation of one's performance [34]. We have chosen to evaluate differences in general self-efficacy rather than parenting specific efficacy because it is this broad construct that we believe will be promoted by the POWs.

The aim of the ELSIPS trial is to provide much needed high quality evidence of the effect of individualised support provided by a lay worker (in this instance a POW), working alongside community midwifery teams, to encourage engagement of nulliparous women with identified social risk factors and the effect on health related outcomes for both mother and baby. It is anticipated that results will be available in the summer of 2013.

## Abbreviations

BEN: Birmingham East and North; BMI: Body mass Index; CLAHRC: Collaborations for Leadership in Applied Health Research and Care; DNA: Did Not Attend; ELSIPS: Evaluation of Lay Support In Pregnant women with Social Risk; EPDS: Edinburgh Postnatal Depression Scale; FNP: Family Nurse Partnership; FSW: Family Support Worker; HOB: Heart of Birmingham; IMD: Index of Multiple Deprivation; LBW: Low birth Weight; MREC: Multicentre Research Ethics Committee; MW: Midwife; NASS: National Asylum Support Service; NFA: No Fixed Abode; NHS: National Health Service; NICE: National Institute for Health and Clinical Excellence; NIHR: National Institute for Health Research; NSF: National Service Framework; NVQ: National Vocational Qualification; PCT: Primary Care Trust; POW: Pregnancy Outreach Worker; RCT: Randomised Controlled Trial; SB: South Birmingham; SD: Standard Deviation; SVB: Spontaneous Vaginal Birth; UK: United Kingdom; US: United States (of America); WHO: World Health Organisation

## Competing interests

The authors declare that they have no competing interests.

## Authors' contributions

SK, CM, KJ and LI designed the study in collaboration with the lead NHS Partner (JC). SK has led the project and is responsible for the day to day management and implementation supported by LI and SD. KH is the study statistician and NG designed and led the qualitative component. SK drafted the paper and all authors have seen and approved the manuscript.

## Pre-publication history

The pre-publication history for this paper can be accessed here:

http://www.biomedcentral.com/1471-2393/12/11/prepub
